# A granular approach to improve reproducibility of the echocardiographic assessment of paravalvular regurgitation after TAVI

**DOI:** 10.1007/s10554-016-0947-4

**Published:** 2016-07-27

**Authors:** Mohammad Abdelghani, Ben Ren, Ernest Spitzer, Hiroki Tateishi, Hans Jonker, Marcel L. Geleijnse, Jan G. Tijssen, Robbert J. de Winter, Patrick W. J. C. Serruys, Osama I. I. Soliman

**Affiliations:** 1 Academic Medical Center, University of Amsterdam, Amsterdam, The Netherlands; 2 Cardialysis Clinical Trials Management and Core Laboratories, Westblaak 98, Rotterdam, 3012KM The Netherlands; 3 Thoraxcenter, Erasmus University Medical Center, ‘s-Gravendijkwal 230, 3015CE Rotterdam, The Netherlands; 4 The International Centre for Circulatory Health, NHLI, Imperial College London, London, UK

**Keywords:** Echocardiography, Imaging, Regurgitation, Aortic-valves

## Abstract

Paravalvular leak (PVL) after transcatheter aortic valve implantation (TAVI) is challenging to quantitate. Transthoracic echocardiography (TTE) is the main tool used for the assessment of PVL but is modestly reproducible. We sought to develop a reproducible echocardiographic approach to assess PVL in the post-TAVI setting. Four observers independently analyzed eleven parameters of PVL severity in 50 pre-discharge TTE studies performed after TAVI. The parameters included color-Doppler parameters [jet circumferential extent (CE) and planimetered vena contracta area in the short-axis view and jet breadth and qualitative features in the long-axis views], continuous-wave Doppler parameters [jet velocity time integral (VTI) and pressure half time (PHT)], quantitative Doppler parameters (regurgitation volume and fraction and effective regurgitant orifice area), aortic diastolic flow reversal and valve stent eccentricity. Intraclass correlation coefficient (ICC) and coefficient of variation (CV) for numerical parameters and kappa coefficient (κ) for categorical parameters were calculated for inter- and intra-observer comparisons. Inter-observer ICC was highest and CV lowest for CE (0.88 and 0.36), jet origin breadth (0.82 and 0.39), jet qualitative features in long-axis views (0.87 and 0.26), jet VTI (0.87 and 0.04) and PHT (0.73 and 0.10). Similar results were found in intra-observer comparisons. A 2-step granular approach combining the most reproducible parameters was used to grade PVL by the four observers. Inter-observer agreement was achieved in 86 % of cases (κ = 0.79). Combining color Doppler and continuous wave Doppler parameters in a granular algorithm yields excellent reproducibility of PVL assessment by TTE.

**Electronic supplementary material:**

The online version of this article (doi:10.1007/s10554-016-0947-4) contains supplementary material, which is available to authorized users.

## Introduction

Transcatheter aortic valve implantation (TAVI) is the treatment of choice for inoperable, a recommended alternative to surgery in high-risk, and a potential option in intermediate-risk patients with symptomatic severe aortic stenosis [1].

Paravalvular leak (PVL) is an important limitation of TAVI as compared to surgical valve replacement [2]. Proper annular sizing [3, 4] and the use of more efficient paravalvular sealing technologies [5–8] led to a significant reduction in the incidence of greater than mild PVL. However, mild PVL is still a common complication of the second/third generation transcatheter aortic valves [5–8], and has been linked to worse prognosis [9]. Moreover, as TAVI extends to younger patients, more bicuspid anatomy and native valvular regurgitation will be met increasing the potential risk of PVL [10, 11]. Furthermore, an excellent paravalvular sealing (at least comparable to surgical bioprosthesis) will be a prerequisite before lower risk patients can be offered TAVI as a recommended option.

Recently, PVL has been reported to regress up to 1 year compared with discharge after TAVI with the self-expanding CoreValve [12]. On the other hand, structural deterioration and new onset valve regurgitation are being increasingly reported [13], further emphasizing the importance of reproducible long-term surveillance.

Ironically, data on the incidence [14], the fate [12, 15, 16] and the consequences [9, 12, 17] of PVL tend to be inconsistent reflecting, in part, poor inter- and intra-technique reproducibility of PVL assessment.

We sought to investigate and propose an approach to improve the reproducibility of the echocardiographic assessment of PVL severity.

## Methods

The study protocol has been approved by the institutional review board and all patients provided a written informed consent. The study consisted of three phases. In the first phase, 50 randomly-selected post-TAVI transthoracic 2D echocardiograms were independently analyzed by four cardiologists (BR, ES, MA and OS) of variable experience (in echocardiography; 5–19 years and in analysis of TAVI echocardiograms; 1–10 years) blinded to patients’ clinical and procedural data. A summary (mean ± standard deviation) of the individual-observer measurements is provided in Table S1. In 35 echocardiograms, reread by the same observer (BR) was performed at a median interval of 5 months to investigate intra-observer reproducibility. Eleven parameters of PVL severity (Table 1) were analyzed in accordance with the guidelines of the American Society of Echocardiography (ASE) and the European association of Echocardiography (EAE) for the evaluation of native [18]/prosthetic [19] aortic regurgitation (AR). Regurgitation volume was calculated as the difference between the stroke volumes at the left and right ventricular outflow tracts, derived from left ventricular outflow tract diameter (LVOTd) and velocity time integral (VTI_LVOT_) and right ventricular outflow tract diameter (RVOTd) and VTI (VTI_RVOT_).

**Table 1:** Echocardiographic parameters of PVL severity included in the reproducibility analysis

Parameter	Description
**Color Doppler**	
Parasternal short-axis view	
PVL circumferential extent (%)	PVL jet arc circumferential extent (in degrees as a fraction of 360°) and planimetered area were measured at the plane of the valve stent inflow edge. Care has been exercised to avoid measuring low velocity (laminar) flow and to include the sum of the separate jets, not the paravalvular arc which includes the non-regurgitant space between jets
PVL short-axis area	
Long-axis views	
Jet origin breadth	PVL jet origin breadth is equivalent to the vena contracta of a transvalvular aortic regurgitation. The sum and the average of measurements from the anterior and posterior sides in the PLAX, apical 5-chamber and 3-chamber views (6 sites in total) were calculated. Ratio of the average jet breadth to the LVOT height was used in the final grading of PVL severity
Qualitative jet features	From the 6 long-axis sites, jet features were assessed and a score (0-none, 1-trace or 2-significant) was accordingly given to the PVL jet; where a jet score of "2" indicates a sizable jet width with continuous turbulence from jet origin to valve stent inflow edge.
**Aortic flow (Pulsed-wave Doppler)**	
Diastolic flow reversal	Descending thoracic and abdominal aortic diastolic flow reversal was sought for by pulsed-wave Doppler from suprasternal and subcostal views, respectively. The duration and end-diastolic velocity of reversed flow were measured. Diastolic flow reversal was subsequently categorized into none-brief, intermediate or holodiastolic-prominent [18]
**Quantitative Doppler**	
Regurgitation volume	RV = SV_LVOT_− SV_RVOT_, where SV = π(diameter/2)^2^ × VTI
Regurgitation fraction	RF = RV/SV_LVOT_ × 100 %
EROA	EROA = RV/VTI_AR_
**Hemodynamic (CWD)**	
VTI_AR_	From apical 5-chamber or 3-chamber view. Choice of either views was based on the image quality and reliability of measurement (complete modal velocity envelop and less variability between cardiac cycles)
Pressure half time	
**Supportive (structural) features**	
Valve stent eccentricity	From the PSAX view, D_max_ and D_min_ of the valve stent were measured in diastole. Eccentricity index = 100 × (D_max_− D_min_)/D_max_

*AR* aortic regurgitation, *CWD* continuous-wave Doppler, *EROA* effective regurgitant orifice area, *LVOT* left ventricular outflow tract, *PLAX* parasternal long-axis, *PSAX* parasternal short-axis, *PVL* paravalvular leak, *PWD* pulsed-wave Doppler, *RF* regurgitation fraction, *RV* regurgitation volume, *RVOT* right ventricular outflow tract, *SV* stroke volume, *VTI* velocity–time integral

In the second phase, data on the inter- and intra-observer reproducibility of the individual parameters were used to generate a reproducible PVL grading scheme. Parameters with the best inter- and intra-observer agreement and the least variability were chosen.

In the third phase, PVL severity was graded by the four observers in the 50 echocardiograms using the tailored scheme. The latter combined several qualitative and semiquantitative parameters of PVL severity. The qualitative features were initially used to categorize patients into clear none-trace PVL, clear severe PVL or an intermediate category. In cases in the intermediate zone, we used three semiquantitative parameters to allocate patients into one of four “granular” [20] sub-classes; mild, mild-to-moderate, moderate, and moderate-to-severe. The latter were then collapsed into two classes (mild and moderate) yielding a 4-class (none-trace, mild, moderate, and severe) final scale. We used the cut-points defined by the ASE/EAE guidelines [18], and experts’ consensus [21] and opinion [22]. In the first 15 studies, independent assessment by the four observers was routinely followed by a consensus grading to align the interpretation of qualitative parameters. More than 1-class disagreement (across the 6 subclasses) in the independent assessments occurred only in two cases. Those 15 cases were subsequently excluded from statistical analysis which was confined to 35 independently-adjudicated cases. Echocardiographic studies varied in image quality but were adequate for grading of PVL (no transvalvular regurgitation was observed), using at least two parameters of severity.

### Statistical analysis

Continuous variables are summarized as mean ± standard deviation (SD) and categorical variables as frequency/percentage of the studied group. Intra- and inter-observer agreement of numerical parameters was expressed as intraclass correlation coefficient (ICC). For inter-observer ICC, pairwise comparisons of the four observers (6 comparisons) were averaged. The *p* value for the averaged ICC was determined according to the degree of freedom (number of pairs). Intra and inter-observer variability was expressed as a coefficient of variation (CV) calculated as the SD of inter-/intra-observer difference divided by the population mean. For intra-observer rereads, differences were the result of subtraction of the second from the first observation. For inter-observer comparisons, the differences were the result of the subtraction of the average observation (ȳ_j_) from the individual observation (y_ij_). Differences among observers were plotted using the method proposed by Jones et al. [23] for graphical assessment of agreement with the mean between multiple observers. In this method; d_ij_ = y_ij_− ȳ_j_ (y-axis) is plotted against y_j_ (x-axis) where y refers to the measurements, ȳ refers to the mean measurement, i refers to observers and j refers to subjects (so ȳ_j_ is the mean of the measurements for subject j). The 95 % limits of agreement (95 % LOA) with the mean are estimated as ±1.96 × s, where s is an estimate of the SD of interobserver differences (for the four observers) and is calculated as the square root of the variance of differences.

Inter-observer and intra-observer agreement on categorical parameters and inter-observer agreement on the PVL grade were expressed as kappa coefficient (κ).

Statistical analysis was performed with SPSS 23 (IBM, Armonk, NY, USA). All probability values were two-tailed, and a *p* value <0.05 was considered significant.

## Results

**Fig. 1:**
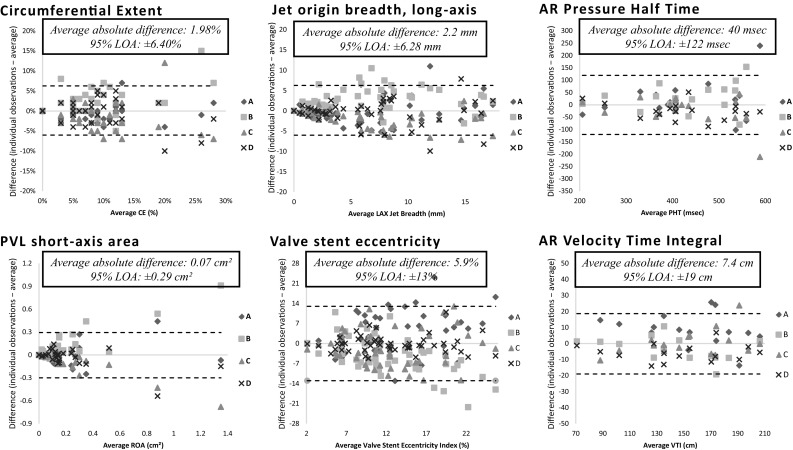
Modified Bland–Altman plots of inter-observer (4 observers; *A, B, C* and *D*) variability and limits of agreement for PVL jet circumferential extent, breadth, short-axis area, pressure half time and velocity time integral and valve stent eccentricity. As visually displayed in the plots, absolute differences (between the individual measurements and the average of all measurements) tended to increase proportionately with increasing average of the measurements (on the X-axis). *AR* aortic regurgitation, *CV* coefficient of variation, *LOA* limit of agreement, *PVL* paravalvular leak, *ROA* regurgitant orifice area

Inter- and intra-observer ICC was high (0.73–0.99) and CV was low (0.01–0.47) for color Doppler parameters (except PVL short-axis area) and continuous-wave Doppler parameters (Tables 2, 3; Fig. 1).

**Table 2:** Indices of inter-observer variability and agreement for eleven parameters of PVL severity

	Inter-observer reproducibility (n = 50 echocardiograms, 4 observers)			
	Average of observations	Average (SD) absolute difference	CV	ICC*
**Color Doppler**				
Parasternal short-axis view				
Circumferential extent	7.0 %	1.98 (2.53) %	0.36	0.88
PVL short-axis area	0.15 cm^2^	0.07 (0.12) cm^2^	0.86	0.81
Long-axis views				
Total jet neck breadth	5.9 mm	2.24 (2.29) mm	0.39	0.82
Qualitative jet features	3.4/12	0.91 (0.89)/12	0.26	0.87
**Continuous-wave Doppler**				
VTI_AR_	149 cm	7.40 (5.91) cm	0.04	0.87
Pressure half time	432 ms	40.4 (43.23) ms	0.10	0.73
**Aortic flow (Pulsed-wave Doppler)**				
Diastolic flow reversal				0.46^§^
**Quantitative Doppler**				
Regurgitation volume	16 ml	12.86 (10.71) ml	0.67	0.59
Regurgitation fraction	20 %	17.23 (16.35) %	0.82	0.61
EROA	0.13 cm^2^	0.08 (0.07) cm^2^	0.54	0.47
**Supportive (structural)**				
Valve stent eccentricity index	12.4 %	5.92 (4.41) %	0.35	0.32

*CV* coefficient of variation, *ICC* intra-class correlation coefficient. Other abbreviations as in Table 1

*p < 0.05, except for EROA (p = 0.23)

^§^Kappa coefficient

**Table 3:** Indices of intra-observer variability and agreement for parameters of PVL severity

	Intra-observer reproducibility (n = 35 echocardiograms, 1 observer)			
	Average of observations	Average (SD) absolute difference	CV	ICC*
**Color Doppler**				
Parasternal short-axis view				
Circumferential extent	5.87 %	2.01 (2.34) %	0.40	0.91
PVL short-axis area	0.11 cm^2^	0.08 (0.07) cm^2^	0.64	0.77
Long-axis views				
Total jet neck breadth	5.47 mm	2.55 (2.56) mm	0.47	0.80
Qualitative jet features	3.91/12	0.99 (1.36)/12	0.35	0.86
**Continuous-wave Doppler**				
VTI_AR_	155.86 cm	1.92 (1.54) cm	0.01	0.99
Pressure half time	444.26 ms	53.02 (37.34) ms	0.08	0.80
**Aortic flow (Pulsed-wave Doppler)**				
Diastolic flow reversal				0.50^§^
**Quantitative Doppler**				
Regurgitation volume	12.11 ml	10.88 (9.78) ml	0.79	0.74
Regurgitation fraction	16.6 %	16.1 (15.02) %	0.90	0.68
**Supportive (structural)**				
Valve stent eccentricity index	10.8 %	4.79 (4.64) %	0.42	0.82

*CV* coefficient of variation, *ICC* intra-class correlation coefficient. Other abbreviations as in Table 1

*p < 0.05, except for RF (p = 0.06)

^§^Kappa coefficient

**Fig. 2:**
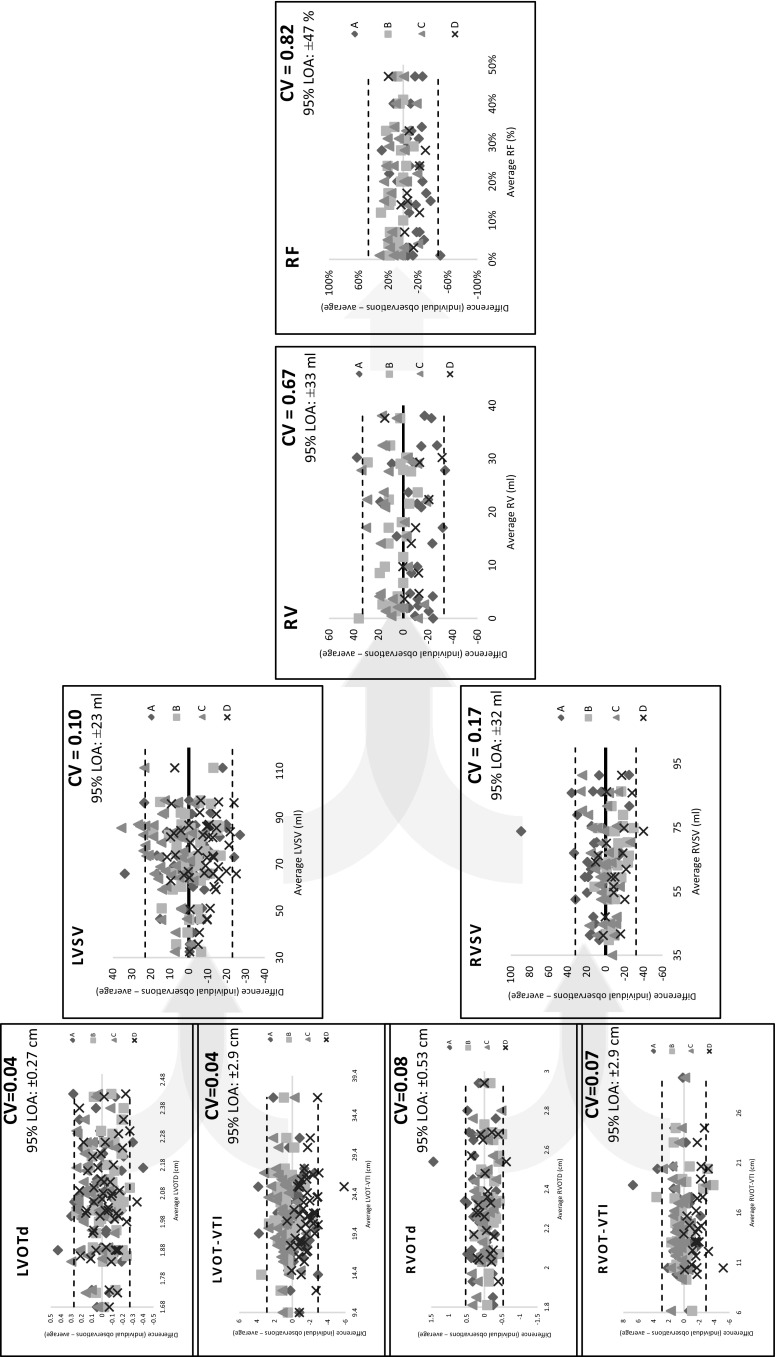
Modified Bland–Altman plots of inter-observer (4 observers; *A, B, C* and *D*) variability and limits of agreement of quantitative Doppler parameters of PVL severity. Variability increased (higher CV and wider 95 % LOA) as basic measurements are subjected to imputations. *CV* coefficient of variation, *LOA* limit of agreement, *LVOTd* left ventricular outflow tract diameter, *LVSV* stroke volume at the left ventricular outflow tract, *RF* regurgitation fraction, *RV* regurgitation volume, *RVOTd* right ventricular outflow tract diameter, *RVSV* stroke volume at the right ventricular outflow tract, *VTI* velocity time interval

Quantitative Doppler parameters, PVL short-axis area and valve stent eccentricity index had lower ICC and higher CV. For quantitative Doppler parameters, the inter-observer CV was generally low for the individual measurements including LVOTd (0.04), VTI_LVOT_ (0.04), RVOTd (0.08), VTI_RVOT_ (0.07), and VTI_AR_ (0.04). Variability, however, markedly increased when computations were applied to calculate LVOT stroke volume (CV = 0.16), RVOT stroke volume (CV = 0.25), effective regurgitant orifice area (CV = 0.54), regurgitation volume (CV = 0.67) and fraction (CV = 0.82) (Fig. 2). Kappa coefficient for aortic diastolic flow reversal was low for inter- (κ = 0.25) and intra-observer (κ = 0.5) comparisons (p > 0.05 for both).

Based on the reproducibility of the individual parameters, the grading scheme (Table 4) was set up and combined six qualitative and three semiquantitative reproducible parameters.

**Table 4:**
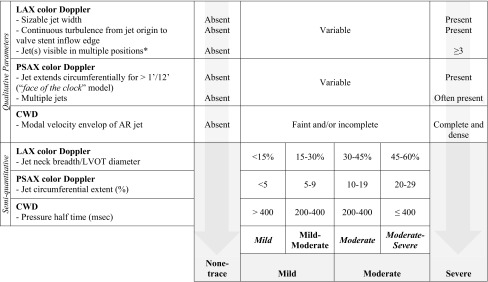
The final PVL grading scheme set-up after considering the reproducibility of the individual parameters

*Two positions in each of the three long-axis views (PLAX, A5C and A3C). Abbreviations as in Table 1

Table S2 shows the number of patients in each of the PVL grades as defined by the four observers using this scheme. Inter-observer grade agreement was achieved in 86 % of cases with a kappa coefficient of 0.79 (Table 5).

**Table 5:** Inter-observer agreement on PVL grade*

(n = 35)	Grade agreement, n (%)	Kappa coefficient^§^
A vs. B	32 (91)	0.865
A vs. C	30 (86)	0.773
A vs. D	31 (89)	0.822
B vs. C	29 (83)	0.728
B vs. D	30 (86)	0.780
C vs. D	30 (86)	0.778
**Average**	**30 (86)**	**0.791**

*As defined using the scheme in Table 2

^§^p < 0.001 for all comparisons

## Discussion

The main findings of the present study are that: (1) color Doppler and continuous wave Doppler parameters are more reproducible than other parameters of PVL severity, especially those entailing complex computations (quantitative Doppler); and that (2) a simplified 2-step granular scheme combining reproducible qualitative and semiquantitative parameters improves the inter-observer reproducibility of PVL grading.

The reported rates of PVL in different TAVI trials and registries ranged from 40 to 67 % for trivial to mild and from 7 to 27 % for moderate to severe AR [14, 24]. In recently published data from a large series treated with a balloon-expandable valve, the incidence of moderate-severe PVL was reported to be 27 % [24]; more than twofolds the incidence reported in former clinical trials utilizing the same valve technology [2]. Those discrepancies are largely to blame on the low reproducibility of the currently used methods to quantitate PVL.

In a random sample from the PARTNER trial, a highly-confident grading of PVL was possible in 62 % of studies, while it was low/uninterpretable in 13 % [20]. In spite of applying different approaches (one that heavily weighs jet circumferential extent vs. a multiparametric multi-window approach) and schemes (condensed vs. granular classification), interobserver PVL grade agreement (39–61 %) and weighted kappa estimates (0.48–0.52) were modest [20].

Our approach was to first investigate the reproducibility of the individual parameters to set-up a scheme that combines the most reproducible ones. To improve practicality, quick qualitative features were primarily used to broadly categorize patients. Afterwards, reproducible semiquantitative parameters were applied in a granular manner. The latter concept (granular classification) was previously shown to improve reproducibility of PVL grading and can easily be collapsed into the ordinary 4-class scheme [20]. The latter is more familiar to the clinicians to interpret and more aligned with other techniques (e.g. angiography and magnetic resonance imaging). This approach resulted in an inter-observer agreement on the PVL grade in 86 % of cases, 0 % greater than 1-grade disagreement and a kappa statistic of 0.79, denoting an excellent reproducibility [25].

Color and continuous-wave Doppler parameters showed favorable reproducibility, while aortic flow and quantitative Doppler parameters were less reproducible. Altiok et al. [26] reported intra- and inter-observer variability of 73.5 ± 52.2 and 108 ± 64.7 % for regurgitation volume and 75.2 ± 55.9 and 120.3 ± 62.3 % for regurgitation fraction of post-TAVI PVL. Noteworthy, in the present study, the component basic measurements of quantitative Doppler criteria showed good reproducibility. Variability, however, overinflated as imputations were applied and increased as imputations were more complex (Fig. 2).

It is widely believed, with little supportive evidence, that the hemodynamics of post-TAVI AR are different from that of chronic native AR [27]. Accordingly, the use of Doppler parameters sensitive to hemodynamics (including CWD parameters) in the assessment of post-TAVI AR is subject to experts’ criticism. On the other hand, two arguments supporting the use of CWD are worth-discussing. First; is that available data supports the correlation between the invasively measured transvalvular diastolic pressure gradient and patients’ outcomes [28]. Second; is that an index that accounts for the hemodynamics on either side of the aortic valve (stiff aorta and small stiff ventricle) should more accurately reflect the hemodynamic significance of an AR jet. It is therefore more relevant to set-up TAVI-specific cut-points of pressure half time as a hemodynamic index of AR severity than precluding its use. Specific cut-points of severity (reflecting the different hemodynamics of PVL from chronic native AR) were thus adopted in the present analysis, but are yet to be further validated.

An interesting counterintuitive finding of the present analysis is that intra-observer agreement and variability were too close to the inter-observer comparisons for most parameters. Similarly, results were quite similar for the four observers despite the wide range of experience. Both findings indicate that the variability reported here is inherent to the parameters of interest with minimal influence of the setting of analysis.

### Limitations

All included echocardiographic studies involved a self-expanding transcatheter aortic valve. Although applicable to other valve types, the findings should be generalized with caution. The cut-points used in classifying the severity of PVL are inadequately validated in TAVI patients [21]. Accuracy of those parameters is, however, beyond the scope of the present study.

## Conclusion

Reproducibility of PVL assessment by transthoracic echocardiography can be improved by using a simplified approach combining reproducible color and continuous wave Doppler parameters.

## Electronic supplementary material

Below is the link to the electronic supplementary material.

Supplementary material 1 (DOCX 15 KB)

## References

[1] Cribier A, Durand E, Eltchaninoff H (2014). Patient selection for TAVI in 2014: is it justified to treat low- or intermediate-risk patients? The cardiologist’s view. EuroIntervention.

[2] Hahn RT, Pibarot P, Stewart WJ, Weissman NJ, Gopalakrishnan D, Keane MG (2013). Comparison of transcatheter and surgical aortic valve replacement in severe aortic stenosis: a longitudinal study of echocardiography parameters in cohort A of the PARTNER trial (placement of aortic transcatheter valves). J Am Coll Cardiol.

[3] Jilaihawi H, Kashif M, Fontana G, Furugen A, Shiota T, Friede G (2012). Cross-sectional computed tomographic assessment improves accuracy of aortic annular sizing for transcatheter aortic valve replacement and reduces the incidence of paravalvular aortic regurgitation. J Am Coll Cardiol.

[4] Hayashida K, Bouvier E, Lefevre T, Hovasse T, Morice MC, Chevalier B (2012). Impact of CT-guided valve sizing on post-procedural aortic regurgitation in transcatheter aortic valve implantation. EuroIntervention.

[5] Wendt D, Al-Rashid F, Kahlert P, Eissmann M, El-Chilali K, Janosi RA (2015). Low incidence of paravalvular leakage with the balloon-expandable Sapien 3 transcatheter heart valve. Ann Thorac Surg.

[6] Schofer J, Colombo A, Klugmann S, Fajadet J, DeMarco F, Tchetche D (2014). Prospective multicenter evaluation of the direct flow medical transcatheter aortic valve. J Am Coll Cardiol.

[7] Manoharan G, Walton AS, Brecker SJ, Pasupati S, Blackman DJ, Qiao H (2015). Treatment of symptomatic severe aortic stenosis with a novel resheathable supra-annular self-expanding transcatheter aortic valve system. JACC Cardiovasc Interv.

[8] Wohrle J, Gonska B, Rodewald C, Trepte U, Koch S, Scharnbeck D (2015). Transfemoral aortic valve implantation with the repositionable Lotus valve compared with the balloon-expandable Edwards Sapien 3 valve. Int J Cardiol.

[9] Kodali S, Pibarot P, Douglas PS, Williams M, Xu K, Thourani V (2015). Paravalvular regurgitation after transcatheter aortic valve replacement with the Edwards sapien valve in the PARTNER trial: characterizing patients and impact on outcomes. Eur Heart J.

[10] Roy DA, Schaefer U, Guetta V, Hildick-Smith D, Mollmann H, Dumonteil N (2013). Transcatheter aortic valve implantation for pure severe native aortic valve regurgitation. J Am Coll Cardiol.

[11] Bauer T, Linke A, Sievert H, Kahlert P, Hambrecht R, Nickenig G (2014). Comparison of the effectiveness of transcatheter aortic valve implantation in patients with stenotic bicuspid versus tricuspid aortic valves (from the German TAVI Registry). Am J Cardiol.

[12] Oh JK, Little SH, Abdelmoneim SS, Reardon MJ, Kleiman NS, Lin G (2015). Regression of paravalvular aortic regurgitation and remodeling of self-expanding transcatheter aortic valve: an observation from the CoreValve U.S. pivotal trial. JACC Cardiovasc Imaging.

[13] Mylotte D, Andalib A, Theriault-Lauzier P, Dorfmeister M, Girgis M, Alharbi W (2015). Transcatheter heart valve failure: a systematic review. Eur Heart J.

[14] Athappan G, Patvardhan E, Tuzcu EM, Svensson LG, Lemos PA, Fraccaro C (2013). Incidence, predictors, and outcomes of aortic regurgitation after transcatheter aortic valve replacement: meta-analysis and systematic review of literature. J Am Coll Cardiol.

[15] Merten C, Beurich HW, Zachow D, Mostafa AE, Geist V, Toelg R (2013). Aortic regurgitation and left ventricular remodeling after transcatheter aortic valve implantation: a serial cardiac magnetic resonance imaging study. Circ Cardiovasc Interv.

[16] Gilard M, Eltchaninoff H, Iung B, Donzeau-Gouge P, Chevreul K, Fajadet J (2012). Registry of transcatheter aortic-valve implantation in high-risk patients. N Engl J Med.

[17] Makkar RR, Fontana GP, Jilaihawi H, Kapadia S, Pichard AD, Douglas PS (2012). Transcatheter aortic-valve replacement for inoperable severe aortic stenosis. N Engl J Med.

[18] Zoghbi WA, Enriquez-Sarano M, Foster E, Grayburn PA, Kraft CD, Levine RA (2003). Recommendations for evaluation of the severity of native valvular regurgitation with two-dimensional and Doppler echocardiography. J Am Soc Echocardiogr.

[19] Zoghbi WA, Chambers JB, Dumesnil JG, Foster E, Gottdiener JS, Grayburn PA (2009). Recommendations for evaluation of prosthetic valves with echocardiography and Doppler ultrasound: a report from the American Society of Echocardiography’s Guidelines and Standards Committee and the Task Force on Prosthetic Valves, developed in conjunction with the American College of Cardiology Cardiovascular Imaging Committee, Cardiac Imaging Committee of the American Heart Association, the European Association of Echocardiography, a registered branch of the European Society of Cardiology, the Japanese Society of Echocardiography and the Canadian Society of Echocardiography, endorsed by the American College of Cardiology Foundation, American Heart Association, European Association of Echocardiography, a registered branch of the European Society of Cardiology, the Japanese Society of Echocardiography, and Canadian Society of Echocardiography. J Am Soc Echocardiogr.

[20] Hahn RT, Pibarot P, Weissman NJ, Rodriguez L, Jaber WA (2015). Assessment of paravalvular aortic regurgitation after transcatheter aortic valve replacement: intra-core laboratory variability. J Am Soc Echocardiogr.

[21] Kappetein AP, Head SJ, Genereux P, Piazza N, van Mieghem NM, Blackstone EH (2012). Updated standardized endpoint definitions for transcatheter aortic valve implantation: the Valve Academic Research Consortium-2 consensus document (VARC-2). Eur J Cardiothorac Surg.

[22] Pibarot P, Hahn RT, Weissman NJ, Monaghan MJ (2015). Assessment of paravalvular regurgitation following TAVR: a proposal of unifying grading scheme. JACC Cardiovasc Imaging.

[23] Jones M, Dobson A, O’Brian S (2011). A graphical method for assessing agreement with the mean between multiple observers using continuous measures. Int J Epidemiol.

[24] Mihara H, Shibayama K, Jilaihawi H, Itabashi Y, Berdejo J, Utsunomiya H (2015). Assessment of post-procedural aortic regurgitation after TAVR: an intraprocedural TEE study. JACC Cardiovasc Imaging.

[25] Viera AJ, Garrett JM (2005). Understanding interobserver agreement: the kappa statistic. Fam Med.

[26] Altiok E, Frick M, Meyer CG, Al Ateah G, Napp A, Kirschfink A (2014). Comparison of two- and three-dimensional transthoracic echocardiography to cardiac magnetic resonance imaging for assessment of paravalvular regurgitation after transcatheter aortic valve implantation. Am J Cardiol.

[27] Gotzmann M, Lindstaedt M, Mugge A (2012). From pressure overload to volume overload: aortic regurgitation after transcatheter aortic valve implantation. Am Heart J.

[28] Sinning JM, Hammerstingl C, Vasa-Nicotera M, Adenauer V, Lema Cachiguango SJ, Scheer AC (2012). Aortic regurgitation index defines severity of peri-prosthetic regurgitation and predicts outcome in patients after transcatheter aortic valve implantation. J Am Coll Cardiol.

