# Supplementary material for: A granular approach to improve reproducibility of the echocardiographic assessment of paravalvular regurgitation after TAVI
Source: Int J Cardiovasc Imaging. 2016 Jul 27;32(10):1519–27. doi: 10.1007/s10554-016-0947-4 (PMC5021735; doi:10.1007/s10554-016-0947-4)
Supplement: Supplementary file 1 — Supplementary material 1 (DOCX 15 KB) [file 10554_2016_947_MOESM1_ESM.docx]

**Supplemental Tables**

**Table S1. Distribution of the PVL severity parameters for each of the four observers (A, B, C and D,** *n=50 echocardiograms***):**

| Parameters | Observer A | Observer B | Observer C | Observer D |
| --- | --- | --- | --- | --- |
| Circumferential extent (%) | 0.07(0.07) | 0.10(0.09) | 0.06(0.07) | 0.08(0.07) |
| PVL short-axis area (cm²) | 0.14(0.29) | 0.27(0.43) | 0.09(0.14) | 0.16(0.22) |
| Total jet neck breadth (mm) | 5.65(6.51) | 8.35(6.50) | 4.00(4.54) | 5.48(5.74) |
| Qualitative jet features | 3.17(2.64) | 4.55(2.63) | 2.62(2.31) | 3.54(2.75) |
| AR VTI (cm) | 164.06(36.80) | 142.19(39.42) | 155.42(37.06) | 130.18(49.46) |
| Pressure half time (msec) | 443.88(148.85) | 457.06(129.21) | 402.78(96.23) | 400.12(98.87) |
| RV (ml) | 20.47(16.76) | 22.11(13.28) | 26.74(18.00) | 11.66(16.00) |
| RF (%) | 25(16) | 29(14) | 29(17) | 17(20) |
| EROA (cm²) | 0.14(0.12) | 0.16(0.08) | 0.19(0.16) | 0.07(0.03) |
| Valve stent eccentricity index (%) | 15.40(9.41) | 17.08(10.56) | 8.27(5.53) | 10.53(8.57) |

Data presented as mean (SD).

Abbreviations as in table 1.

**Table S2.** The severity of PVL as defined by the four observers (n=35).

|  | **A** | **B** | **C** | **D** |
| --- | --- | --- | --- | --- |
| **None-to-trace** | 13 | 11 | 12 | 14 |
| **Mild** | 16 | 17 | 17 | 14 |
| **Moderate** | 5 | 6 | 5 | 6 |
| **Severe** | 1 | 1 | 1 | 1 |
